# Similarities and Differences in COVID-19 Awareness, Concern, and Symptoms by Race and Ethnicity in the United States: Cross-Sectional Survey

**DOI:** 10.2196/20001

**Published:** 2020-07-10

**Authors:** Jeb Jones, Patrick S Sullivan, Travis H Sanchez, Jodie L Guest, Eric W Hall, Nicole Luisi, Maria Zlotorzynska, Gretchen Wilde, Heather Bradley, Aaron J Siegler

**Affiliations:** 1 Department of Epidemiology Rollins School of Public Health Emory University Atlanta, GA United States; 2 Department of Behavioral, Social and Health Education Sciences Rollins School of Public Health Emory University Atlanta, GA United States; 3 Department of Epidemiology & Biostatistics School of Public Health Georgia State University Atlanta, GA United States

**Keywords:** COVID-19, SARS-CoV-2, race, ethnicity, awareness, concern, symptom, cross-sectional, knowledge, health disparity, inequality

## Abstract

**Background:**

Existing health disparities based on race and ethnicity in the United States are contributing to disparities in morbidity and mortality during the coronavirus disease (COVID-19) pandemic. We conducted an online survey of American adults to assess similarities and differences by race and ethnicity with respect to COVID-19 symptoms, estimates of the extent of the pandemic, knowledge of control measures, and stigma.

**Objective:**

The aim of this study was to describe similarities and differences in COVID-19 symptoms, knowledge, and beliefs by race and ethnicity among adults in the United States.

**Methods:**

We conducted a cross-sectional survey from March 27, 2020 through April 1, 2020. Participants were recruited on social media platforms and completed the survey on a secure web-based survey platform. We used chi-square tests to compare characteristics related to COVID-19 by race and ethnicity. Statistical tests were corrected using the Holm Bonferroni correction to account for multiple comparisons.

**Results:**

A total of 1435 participants completed the survey; 52 (3.6%) were Asian, 158 (11.0%) were non-Hispanic Black, 548 (38.2%) were Hispanic, 587 (40.9%) were non-Hispanic White, and 90 (6.3%) identified as other or multiple races. Only one symptom (sore throat) was found to be different based on race and ethnicity (*P*=.003); this symptom was less frequently reported by Asian (3/52, 5.8%), non-Hispanic Black (9/158, 5.7%), and other/multiple race (8/90, 8.9%) participants compared to those who were Hispanic (99/548, 18.1%) or non-Hispanic White (95/587, 16.2%). Non-Hispanic White and Asian participants were more likely to estimate that the number of current cases was at least 100,000 (*P*=.004) and were more likely to answer all 14 COVID-19 knowledge scale questions correctly (Asian participants, 13/52, 25.0%; non-Hispanic White participants, 180/587, 30.7%) compared to Hispanic (108/548, 19.7%) and non-Hispanic Black (25/158, 15.8%) participants.

**Conclusions:**

We observed differences with respect to knowledge of appropriate methods to prevent infection by the novel coronavirus that causes COVID-19. Deficits in knowledge of proper control methods may further exacerbate existing race/ethnicity disparities. Additional research is needed to identify trusted sources of information in Hispanic and non-Hispanic Black communities and create effective messaging to disseminate correct COVID-19 prevention and treatment information.

## Introduction

A novel coronavirus, severe acute respiratory syndrome 2 (SARS-CoV-2), was identified in Wuhan, China, in December 2019; the virus quickly spread worldwide and was labeled a pandemic by the World Health Organization on March 11, 2020 [1]. SARS-CoV-2 can result in severe respiratory infection, causing coronavirus disease (COVID-19). The rapid spread and high estimated infectivity of SARS-CoV-2 coupled with the severity of COVID-19 led to widespread shuttering of businesses and implementation of mandatory stay-at-home orders across the United States. Although reported cases are not stratified by race and ethnicity in all US jurisdictions or may be incompletely reported due to missing information, available data indicate the emergence of racial and ethnic disparities in the occurrence of COVID-19 [2], mirroring disparities that have been well characterized in other disease processes.

Racial and ethnic disparities in COVID-19 occurrence may be due to multiple factors. Asian, Black, and Hispanic Americans are all more likely to be uninsured than non-Hispanic White Americans [3], resulting in reduced access to health care. Increased prevalence of preexisting conditions that are known to be associated with poor outcomes in COVID-19 patients, including diabetes [4,5] among Black and Hispanic people and hypertension [5-7] among Black people, may increase susceptibility and disease severity in these populations. The long-term effects of structural racism and income inequality that cause larger proportions of people of color to work in conditions that are not conducive to social distancing [8] or to live in crowded conditions [8,9] also facilitate efficient transmission of the virus. In addition to combatting these existing disparities, in a rapidly evolving pandemic, accurate information is necessary for individuals to exercise appropriate prevention and care-seeking behavior. We examined whether there were racial or ethnic differences in knowledge, stigma, and experience of symptoms during the early stages of the COVID-19 pandemic.

## Methods

Advertisements on Facebook, Snapchat, and Twitter were used to conduct a nationwide web-based survey with US adults aged 18 years or older from March 27 through April 1, 2020 [10]. Participants were eligible to participate if they were at least 18 years of age. We sought to obtain a racially and ethnically diverse sample by overrecruiting people of color and Hispanic participants. Therefore, because a sufficient number of non-Hispanic White participants had been enrolled, enrollment of non-Hispanic White participants was stopped on the final day of recruiting to increase the representation of communities of color in the survey. Participants were not compensated for completing the survey. All study procedures were reviewed and approved by the Emory University Institutional Review Board.

The survey included questions on knowledge of effective prevention methods, a COVID-19 stigma scale, and current COVID-19–related symptoms (see Multimedia Appendix 1). We used chi-square tests with Holm Bonferroni correction to account for multiple comparisons to assess differences across these domains based on race and ethnicity (Asian, Hispanic, non-Hispanic Black, non-Hispanic White, and other/multiple race). Corrected *P* values <.05 were considered statistically significant. The full survey is included in Multimedia Appendix 1. All analyses were conducted using SAS 9.4 (SAS Institute).

## Results

A total of 1435 participants consented to participate and completed the survey. Of the 1435 participants, 52 (3.6%) were Asian, 548 (38.2%) were Hispanic, 158 (11.0%) were non-Hispanic Black, 587 (40.9%) were non-Hispanic White, and 90 (6.3%) were of other or multiple races. There were race and ethnicity differences in gender, annual income, educational status, and geographic region of residence across categories of race and ethnicity (Table 1).

When asked whether they were currently experiencing symptoms possibly related to COVID-19, a plurality of participants (635/1435, 44.3%) reported no symptoms (Table 2). Of those experiencing COVID-19 symptoms, the most frequently reported symptoms were cough (328/1435, 22.9%), sneezing (322/1435, 22.4%), and headache (311/1435, 21.7%). The only symptom that differed by race/ethnicity was sore throat. Racial/ethnic differences were observed in perceived likelihood of currently having COVID-19 by race/ethnicity. Hispanic (192/544, 35.3%) and non-Hispanic Black (54/157, 34.4%) participants were more likely to report that it was somewhat likely, likely, or very likely that they currently had COVID-19 compared to Asian (11/51, 21.6%) and non-Hispanic White (121/580, 20.9%) participants.

When asked about their knowledge of numbers of cases and COVID-19 deaths, non-Hispanic White (376/587, 64.1%) and Asian (32/52, 61.5%) participants were more likely to correctly estimate that there were currently 100,000 or more cases in the US compared to Hispanic (269/548, 49.1%) and non-Hispanic Black (73/158, 46.2%) participants. No differences were observed in the estimated number of deaths expected from COVID-19 by the end of 2020.

Non-Hispanic White (180/587, 30.7%) and Asian (13/52, 25.0%) participants were more likely to answer all 14 COVID-19 knowledge scale questions correctly compared to Hispanic (108/548, 19.7%) and non-Hispanic Black (25/158, 15.8%) participants. Overall, 46.6% of participants endorsed at least one stigmatizing statement related to COVID-19, with no difference by race.

Overall, 954/1247 participants (76.5%) indicated willingness to participate in future research studies of diagnostic and serologic testing for SARS-CoV-2, with no differences by race and ethnicity.

**Table 1:** Demographic characteristics of participants in a cross-sectional web-based survey of COVID-19 symptoms and knowledge from March 27 through April 1, 2020 (N=1435).

Characteristic		Total (N=1435)	Asian (n=52, 3.6%)	Hispanic (n=548, 38.2%)	Non-Hispanic Black (n=158, 11.0%)	Non-Hispanic White (n=587, 40.9%)	Other/multiple race (n=90, 6.3%)	*P* value^a^
Age (years), median (IQR)		33 (24-57)	25 (21-31)	30 (22-44)	25 (20-38)	54 (31-64)	31 (21-58)	.002
**Gender, n (%)^b^**								.002
	Male	536 (40.2)	20 (41.7)	236 (48.4)	49 (36.8)	202 (34.4)	29 (37.7)	
	Female	761 (57.1)	26 (54.2)	249 (51.0)	83 (62.4)	360 (61.3)	43 (55.8)	
	Other	36 (2.7)	2 (4.2)	3 (0.6)	1 (0.8)	25 (4.3)	5 (6.5)	
**Annual Income (US $), n (%)**								.002
	<30,000	376 (36.1)	9 (25.7)	150 (41.3)	54 (49.1)	139 (29.3)	24 (40.7)	
	30,000-74,999	397 (38.1)	12 (34.3)	127 (35.0)	43 (39.1)	192 (40.5)	23 (39.0)	
	≥75,000	268 (25.7)	14 (40.0)	86 (23.7)	13 (11.8)	143 (30.2)	12 (20.3)	
**Education, n (%)**								.004
	High school or less	202 (16.7)	5 (10.4)	99 (22.9)	15 (11.8)	69 (12.9)	14 (19.4)	
	At least some college	1011 (83.4)	43 (89.6)	333 (77.1)	112 (88.2)	465 (87.1)	58 (80.6)	
**Region, n (%)**								.002
	Midwest	280 (19.6)	7 (13.5)	57 (10.4)	27 (17.1)	177 (30.3)	12 (13.3)	
	Northeast	251 (17.5)	9 (17.3)	82 (15.0)	22 (13.9)	117 (20.0)	9 (17.3)	
	South	540 (37.7)	8 (15.4)	215 (39.4)	86 (54.4)	200 (34.2)	8 (15.4)	
	West	360 (25.2)	28 (53.9)	192 (35.2)	23 (14.6)	91 (15.6)	28 (53.9)	

^a^Holm Bonferroni *P* value to correct for multiple comparisons; results were considered statistically significant if corrected *P*<.05.

^b^Category totals do not sum to the total of 1435 due to missing data.

**Table 2:** Associations between race/ethnicity and symptoms, likelihood of current COVID-19 infection, estimates of the extent of the COVID-19 pandemic, knowledge, stigma, and interest in participating in research studies among participants in a web-based, cross-sectional survey conducted from March 27 through April 1, 2020 (N=1435).

Variable		Total (N=1435), n (%)	Asian (n=52, 3.6%), n (%)	Hispanic (n=548, 38.2%), n (%)	Non-Hispanic Black (n=158, 11.0%), n (%)	Non-Hispanic White (n=587, 40.9%), n (%)	Other/multiple race (n=90, 6.3%), n (%)	*P* value^a^
**Symptoms in last 24 hours**								
	Fever	35 (2.4)	1 (1.9)	10 (1.8)	6 (3.8)	13 (2.2)	5 (5.6)	>.99
	Cough	328 (22.9)	9 (17.3)	119 (21.7)	30 (19.0)	159 (27.1)	11 (12.2)	.07
	Sneezing	322 (22.4)	11 (21.2)	124 (22.6)	37 (23.4)	129 (22.0)	21 (23.3)	>.99
	Sore throat	214 (14.9)	3 (5.8)	99 (18.1)	9 (5.7)	95 (16.2)	8 (8.9)	.003
	Headache	311 (21.7)	10 (19.2)	110 (20.1)	43 (27.2)	127 (21.6)	21 (23.3)	>.99
	Shortness of breath	94 (6.6)	3 (5.8)	31 (5.7)	5 (3.2)	47 (8.0)	8 (8.9)	>.99
	Diarrhea	108 (7.5)	3 (5.8)	42 (7.7)	9 (5.7)	47 (8.0)	7 (7.8)	>.99
	Myalgia	103 (7.2)	2 (3.9)	36 (6.6)	7 (4.4)	54 (9.2)	4 (4.4)	>.99
	Feeling of being unwell	175 (12.2)	8 (15.4)	69 (12.6)	17 (10.8)	70 (11.9)	11 (12.2)	>.99
	No symptoms	635 (44.3)	23 (44.2)	246 (44.9)	75 (47.5)	251 (42.8)	40 (44.4)	>.99
**COVID-19^b^ likelihood^c^**								.002
	Very unlikely	356 (25.0)	8 (15.7)	129 (23.7)	37 (23.6)	157 (27.1)	25 (27.8)	
	Unlikely	661 (46.5)	32 (62.8)	223 (41.0)	66 (42.0)	302 (52.1)	38 (42.2)	
	Somewhat likely	324 (22.8)	9 (17.7)	149 (27.4)	41 (26.1)	103 (17.8)	22 (24.4)	
	Likely	47 (3.3)	2 (3.9)	24 (4.4)	10 (6.4)	9 (1.6)	2 (2.2)	
	Very likely	34 (2.4)	0 (0.0)	19 (3.5)	3 (1.9)	9 (1.6)	3 (3.3)	
**Estimated number of current cases^d^**								.004
	<1000	28 (2.0)	1 (1.9)	14 (2.6)	4 (2.5)	9 (1.5)	0 (0.0)	
	1000-9999	496 (34.6)	17 (32.7)	215 (39.2)	72 (45.6)	157 (26.8)	35 (38.9)	
	10,000-99,999	113 (7.9)	2 (3.9)	50 (9.1)	9 (5.7)	45 (7.7)	7 (7.8)	
	100,000-499,999	458 (31.9)	18 (34.6)	161 (29.4)	43 (27.2)	209 (35.6)	27 (30.0)	
	500,000-999,999	139 (9.7)	3 (5.8)	52 (9.5)	8 (5.1)	69 (11.8)	7 (7.8)	
	≥1,000,000	201 (14.0)	11 (21.2)	56 (10.2)	22 (13.9)	98 (16.7)	14 (15.6)	
**Estimated number of deaths^c^**								>.99
	Fewer than 1000	55 (4.0)	2 (4.0)	20 (3.9)	8 (5.1)	20 (3.6)	5 (6.0)	
	1000-10,000	266 (19.5)	8 (16.0)	82 (15.8)	38 (24.2)	125 (22.6)	13 (15.5)	
	10,001-100,000	478 (35.0)	16 (32.0)	184 (35.4)	54 (34.4)	194 (35.0)	30 (35.7)	
	100,000-1,000,000	430 (31.5)	17 (34.0)	186 (35.8)	48 (30.6)	152 (27.4)	27 (32.1)	
	≥1,000,000	136 (10.0)	7 (14.0)	48 (9.2)	9 (5.7)	63 (11.4)	9 (10.7)	
**Knowledge index**								.002
	<12	418 (29.1)	13 (25.0)	196 (35.8)	61 (38.6)	116 (19.8)	32 (35.6)	
	12-13	675 (47.0)	26 (50.0)	244 (44.5)	72 (45.6)	291 (49.6)	42 (46.7)	
	14	342 (23.8)	13 (25.0)	108 (19.7)	25 (15.8)	180 (30.7)	16 (17.8)	
**Stigma index^c^**								.09
	0	722 (53.4)	31 (62.0)	242 (47.1)	71 (48.6)	329 (59.2)	49 (56.3)	
	1-2	525 (38.8)	16 (32.0)	221 (43.0)	65 (44.5)	191 (34.4)	32 (36.8)	
	≥3	106 (7.8)	3 (6.0)	51 (9.9)	10 (6.9)	36 (6.5)	6 (6.9)	
**Interest in learning about another study^c^**								>.99
	Yes	954 (76.5)	35 (72.9)	352 (78.4)	94 (71.2)	416 (76.5)	57 (77.0)	
	No	293 (23.5)	13 (27.1)	97 (21.6)	38 (28.8)	128 (23.5)	17 (23.0)	

^a^Holm Bonferroni *P* value to correct for multiple comparisons; results were considered statistically significant if corrected *P*<.05.

^b^COVID-19: coronavirus disease.

^c^Category total does not sum to the column total due to missing data.

^d^Values greater than the population of the United States (328 million) were excluded. At the beginning of the study period (March 27, 2020), there were 107,000 cumulative confirmed cases in the United States. At the end of the study period (April 1, 2020), there were 213,400 cumulative confirmed cases in the United States.

## Discussion

### Principal Findings

We observed few differences in prevalent symptoms consistent with COVID-19 among a web-based sample of adults in the United States at the end of March through the beginning of April 2020. With respect to estimating the extent of the epidemic in the United States during the study period, there were 101,700 cumulative confirmed cases and 1600 cumulative deaths as of March 27, 2020 and 213,400 cumulative confirmed cases and 5000 cumulative deaths by April 1, 2020 [11].

Despite the similarity in experiences of COVID-19 symptoms, we did observe differences in participants’ self-assessed likelihood of having COVID-19 at the time of survey completion. Hispanic and non-Hispanic Black participants were more likely to suspect that they were currently infected, which may reflect an awareness of the differential impact of the pandemic on communities of color in the United States or a better understanding of the potential for asymptomatic infection. These differential self-assessments of the likelihood of current infection may translate to differential testing and care-seeking behavior. Trends in self-assessment of symptoms and knowledge of appropriate prevention strategies should be continuously monitored to identify any persistent differences based on race and ethnicity.

### Limitations

This study has limitations. Participants were recruited on the internet via advertisements on social media sites and are not representative of all adults in the United States. Participation in an uncompensated survey may reflect a prior interest in COVID-19; therefore, the respondents may have more COVID-19 knowledge compared to the general population. However, we do not think that this selection bias would be differential by race or ethnicity.

### Conclusions

Racial and ethnic disparities in morbidity and mortality due to COVID-19 are already apparent. Black and Hispanic populations bear a disproportionate burden of medical conditions across their lifespans, including obesity, diabetes, and heart disease [4,5,12]. Worse health outcomes are exacerbated by structural racism, which in turn impacts housing, economic opportunities, education, transportation, food availability, and health care access [13]. Previously existing disparities in chronic disease incidence and prevalence due to unequal access and treatment [14] that are at least partially contributing to the observed COVID-19 disparities could increase if people are relying on incorrect prevention methods. Knowledge gaps in effective prevention methods and an inability to adhere to known best prevention methods due to crowding and work conditions have the potential to exacerbate these underlying disparities.

Addressing the structural racism that results in differential access to care will necessarily involve structural changes to health care delivery [15]. Universal access to COVID-19 testing, treatment, and if available, a SARS-CoV-2 vaccine will be critical but not sufficient. Medical mistrust must also be addressed [16]. Lack of trust in the medical establishment is an artifact of historical mistreatment of populations of color and of misinformation. It will be necessary to identify methods to overcome medical mistrust to ensure acceptance of treatment and prevention measures. Additionally, research is needed to identify trusted sources of information, as well as modes and mechanisms of communication, in Black and Hispanic communities to ensure that correct prevention and treatment information can be disseminated most effectively. Reducing disparities in knowledge, health care access, prevention, and treatment will be necessary conditions for successfully combatting the COVID-19 pandemic.

## Appendix

Multimedia Appendix 1 Full survey.

## Abbreviations

COVID-19: coronavirus disease
SARS-CoV-2: severe acute respiratory syndrome 2

## References

[1] (2020). WHO Director-General's opening remarks at the media briefing on COVID-19 - 11 March 2020. World Health Organization.

[2] Dorn AV, Cooney RE, Sabin ML (2020). COVID-19 exacerbating inequalities in the US. Lancet.

[3] Berchick ER, Barnett JC, Upton RD (2019). Health Insurance Coverage in the United States: 2018 Current Population Reports. United States Census Bureau.

[4] Hussain A, Bhowmik B, do Vale Moreira NC (2020). COVID-19 and diabetes: Knowledge in progress. Diabetes Res Clin Pract.

[5] Balfour PC, Ruiz JM, Talavera GA, Allison MA, Rodriguez CJ (2016). Cardiovascular Disease in Hispanics/Latinos in the United States. J Lat Psychol.

[6] Li B, Yang J, Zhao F, Zhi L, Wang X, Liu L, Bi Z, Zhao Y (2020). Prevalence and impact of cardiovascular metabolic diseases on COVID-19 in China. Clin Res Cardiol.

[7] Louisiana Coronavirus COVID-19. Louisiana Department of Health.

[8] (2020). COVID-19 in Racial and Ethnic Minority Groups. US Centers for Disease Control and Prevention.

[9] Burr JA, Mutchler JE, Gerst K (2010). Patterns of residential crowding among Hispanics in later life: immigration, assimilation, and housing market factors. J Gerontol B Psychol Sci Soc Sci.

[10] Siegler A, Hall E, Luisi N, Zlotorzynska M, Wilde G, Sanchez T (2020). Willingness to seek laboratory testing for SARS-CoV-2 with home, drive-through, and clinic-based specimen collection locations. Open Forum Infect Dis.

[11] COVID-19 United States Cases by County. Johns Hopkins Coronavirus Resource Center.

[12] Wang Y, Beydoun MA (2007). The obesity epidemic in the United States--gender, age, socioeconomic, racial/ethnic, and geographic characteristics: a systematic review and meta-regression analysis. Epidemiol Rev.

[13] Laurencin CT, McClinton A (2020). The COVID-19 Pandemic: a Call to Action to Identify and Address Racial and Ethnic Disparities. J Racial Ethn Health Disparities.

[14] Manuel JI (2018). Racial/Ethnic and Gender Disparities in Health Care Use and Access. Health Serv Res.

[15] Williams DR, Cooper LA (2019). Reducing Racial Inequities in Health: Using What We Already Know to Take Action. Int J Environ Res Public Health.

[16] Jaiswal J, Halkitis PN (2019). Towards a More Inclusive and Dynamic Understanding of Medical Mistrust Informed by Science. Behav Med.

