# Supplementary material for: Similarities and Differences in COVID-19 Awareness, Concern, and Symptoms by Race and Ethnicity in the United States: Cross-Sectional Survey
Source: J Med Internet Res. 2020 Jul 10;22(7):e20001. doi: 10.2196/20001 (PMC7357692; doi:10.2196/20001)
Supplement: Multimedia Appendix 1 [file jmir_v22i7e20001_app1.pdf]

Emory University COVID-19 Study

Intro

Value: [*tracking variable for recruitment source*]

Value: [*system variable for IP address*]

Coronavirus (COVID-19) is an infectious disease that causes respiratory illness.

Emory University is conducting a study to learn about the public's knowledge and perception of the disease, along with willingness to use a home test. You must be at least 18 years of age to participate in the study.

Our privacy policy: Any information shared in this survey is strictly confidential and will only be used for research study purposes. All the information that we gather from you today is safely stored.

Please read the information about the study in the box below and indicate whether or not you would like to participate.

(On a mobile device you may need to scroll to the bottom to see the question.)

Emory University IRB Consent Form for Study "Acceptability survey of home-based testing options for SARS-CoV-2" inserted in scroll box here.

Being in this study is entirely your choice. You have the right to refuse to participate or to stop taking the survey at any time. Please select an option below. You may print a copy of this form for your records.*

- I am at least 18 years of age, agree to the above information and would like to participate in this research study.
- I would not like to continue as a participant in this research study.

Page exit logic: Skip / Disqualify Logic

IF respondent selects "I would not like to continue as a participant in this research study." THEN Disqualify and display: "Sorry, you do not qualify to take this survey. Thank you for your time."

Demographics: Part 1

How old are you?*

[ ]

Do you consider yourself to be Latino/a or of Hispanic origin?*

- Yes
- No

What race are you?*

- Asian/Pacific Islander
- Black/African American
- White/Caucasian
- Native American/Alaska Native
- Mixed Race
- Other (please specify): [ ]

Note: State question was moved from Part 2 Demographics to Part 1 Demographics on the final day of data collection as part of amendment to increase minority representation in the sample.

Where do you live?*

[dropdown menu with US states]

Page exit logic: Skip / Disqualify Logic

IF respondent enters a number less than 18 for "How old are you?" THEN Disqualify and display: "Sorry, you do not qualify to take this survey. Thank you for your time."

Additional logic added on the final day of data collection in an effort to increase minority representation in the sample:

IF respondent enters "White/Caucasian" for "What race are you?" AND "No" for "Do you consider yourself to be Latino/a or of Hispanic origin?" THEN Disqualify and display: "Sorry, you do not qualify to take this survey. Thank you for your time."

COVID-19 Knowledge/Testing/Symptoms

Question adapted from: Geldsetzer P. Knowledge and Perceptions of COVID-19 Among the General Public in the United States and the United Kingdom: A Cross-sectional Online Survey. Ann Intern Med. 2020; [Epub ahead of print 20 March 2020]. doi: https://doi.org/10.7326/M20-0912

Which of the following actions help prevent catching an infection with the new coronavirus?
Please select True or False for each option.

	True	False
Wear a face mask	○	○
Getting a vaccination against pneumonia	○	○
Gargling mouthwash	○	○
Washing your hands	○	○
Eating garlic	○	○
Avoid close contact with people who are sick	○	○
Taking antibiotics	○	○
Using a hand dryer	○	○
Putting sesame oil on your skin	○	○
Avoiding touching your eyes, nose, and mouth with unwashed hands	○	○
Regularly rinsing your nose with saline	○	○

Question adapted from: Geldsetzer P. Knowledge and Perceptions of COVID-19 Among the General Public in the United States and the United Kingdom: A Cross-sectional Online Survey. Ann Intern Med. 2020; [Epub ahead of print 20 March 2020]. doi: https://doi.org/10.7326/M20-0912

Is the following statement true or false?

Consistently wearing a face mask is highly effective in protecting you from getting infected with the new coronavirus.

For the purpose of this question, "highly effective" is defined as reducing your risk of getting infected by >95% and a"face mask" is a common medical mask.

- ◯ True
- ◯ False

Question adapted from: Geldsetzer P. Knowledge and Perceptions of COVID-19 Among the General Public in the United States and the United Kingdom: A Cross-sectional Online Survey. Ann Intern Med. 2020; [Epub ahead of print 20 March 2020]. doi: https://doi.org/10.7326/M20-0912

What is the main way in which people are currently getting infected with the new coronavirus?
Please select one.

- ◯ Eating or touching bats
- ◯ Fecal contaminants in drinking water
- ◯ Unhygienic preparation of food
- ◯ Sexual intercourse or sharing of needles for drug use
- ◯ Mosquito bites
- ◯ Droplets of saliva that land in the mouths or noses of people who are nearby when an infected person sneezes or coughs
- ◯ Eating undercooked meat products
- ◯ Directly coming into touch with someone's bodily fluids like blood, vomit, or sweat
- ◯ Snake bites or touching snakes

Is the following statement true or false?

People who have COVID-19 may transmit the disease without showing symptoms.

- ◯ True
- ◯ False

COVID-19 Stigmatizing Beliefs

Question adapted from: Geldsetzer P. Knowledge and Perceptions of COVID-19 Among the General Public in the United States and the United Kingdom: A Cross-sectional Online Survey. Ann Intern Med. 2020; [Epub ahead of print 20 March 2020]. doi: https://doi.org/10.7326/M20-0912

Uber is a ride-sharing app that allows people to request and pay for a car ride via their phone. The Uber driver then either rejects or accepts the ride request.

If you were an Uber driver today, would you try to reject ride requests from people with Asian-sounding names (or a profile photo of Asian ethnicity) to reduce your risk of getting infected with the new coronavirus?

- Never
- Sometimes
- Often
- Always

Question adapted from: Geldsetzer P. Knowledge and Perceptions of COVID-19 Among the General Public in the United States and the United Kingdom: A Cross-sectional Online Survey. Ann Intern Med. 2020; [Epub ahead of print 20 March 2020]. doi: https://doi.org/10.7326/M20-0912

Does receiving a letter or package from China put you at risk of getting infected with the new coronavirus?

- Yes
- No

Should officials call the new coronavirus the Chinese coronavirus?

- Yes
- No

Is China to blame for COVID-19?

- Yes
- No

COVID-19 Perceived Relevance

Question adapted from: Geldsetzer P. Knowledge and Perceptions of COVID-19 Among the General Public in the United States and the United Kingdom: A Cross-sectional Online Survey. Ann Intern Med. 2020; [Epub ahead of print 20 March 2020]. doi: https://doi.org/10.7326/M20-0912

What do you think is the number of people living in the United States who are infected with the new coronavirus? This number should include both those who have been diagnosed and those who have not been diagnosed.

Question adapted from: Geldsetzer P. Knowledge and Perceptions of COVID-19 Among the General Public in the United States and the United Kingdom: A Cross-sectional Online Survey. Ann Intern Med. 2020; [Epub ahead of print 20 March 2020]. doi: https://doi.org/10.7326/M20-0912

How many people in the US do you think will die from the new coronavirus by the end of 2020?

- ○ 0
- ○ 1 –100
- ○ 101 –500
- ○ 501 –1,000
- ○ 1,001 –10,000
- ○ 10,001 –100,000
- ○ 100,001 –1 million
- ○ Between 1 million and 10 million
- ○ Between 10 million and 30 million
- ○ More than 30 million

COVID-19 Perceived Symptoms

How likely do you think it is that you have COVID-19 right now?

- ○ Very unlikely
- ○ Unlikely
- ○ Somewhat likely
- ○ Likely
- ○ Very likely

Have you had any of the following in the last 24 hours?
Check all that apply.

- ☐ Fever
- ☐ Cough
- ☐ Sneezing

☐ Sore throat

☐ Headache

☐ Shortness of breath

☐ Diarrhea

☐ Myalgia (muscle aches)

☐ Feeling of being unwell

☐ None of these

COVID-19 Home Test Perceptions Part 1

The next questions are about testing for COVID-19 by using a saliva sample or throat swab. Your results would be returned to you electronically and privately.

The following definitions were displayed to respondents in a pop-up box if clicked for more information.

- What is a home saliva sample? A home saliva sample would involve you spitting in a tube and sending it to a certified laboratory.
- What is a home throat swab? A home throat swab would involve you using a throat swab and sending it into a certified laboratory.
- What is a drive-through site? A drive-through site for throat swab would involve your traveling to a drive-through facility in your car to have a healthcare worker collect the swab.
- What is a laboratory throat swab? A laboratory throat swab would involve your traveling to a laboratory facility in a clinic or private laboratory to have a healthcare worker collect the swab.

For a research study:*

	Strongly Agree	Agree	Undecided	Disagree	Strongly Disagree
I would be willing to collect a saliva sample for a home test as part of a research study.	○	○	○	○	○
I would be willing to use a home throat swab test as part of a research study.	○	○	○	○	○
I would be willing to go to a drive-through site for throat swab test as part of a research study.	○	○	○	○	○

I would be willing to go to a clinic for a lab-collected throat swab test as part of a research study.	○	○	○	○	○

COVID-19 Home Test Perceptions Part 2

The next questions are about testing for COVID-19 by using a saliva sample or throat swab. Your results would be returned to you electronically and privately.

The following definitions were displayed to respondents in a pop-up box if clicked for more information.

- What is a home saliva sample? A home saliva sample would involve you spitting in a tube and sending it to a certified laboratory.
- What is a home throat swab? A home throat swab would involve you using a throat swab and sending it into a certified laboratory.
- What is a drive-through site? A drive-through site for throat swab would involve your traveling to a drive-through facility in your car to have a healthcare worker collect the swab.
- What is a laboratory throat swab? A laboratory throat swab would involve your traveling to a laboratory facility in a clinic or private laboratory to have a healthcare worker collect the swab.

To learn if I have COVID-19:*

	Strongly Agree	Agree	Undecided	Disagree	Strongly Disagree
I would be willing to collect a saliva sample for a home test to learn if I have COVID-19.	○	○	○	○	○
I would be willing to use a home throat swab test to learn if I have COVID-19.	○	○	○	○	○
I would be willing to go to a drive-through site for a throat swab test to learn if I have COVID-19.	○	○	○	○	○
I would be willing to go to a clinic for a lab-collected throat swab test to learn if I have COVID-19.	○	○	○	○	○

COVID-19 Home Test Perceptions Part 3

The next questions are about testing for COVID-19 by using a saliva sample or throat swab. Your results would be returned to you electronically and privately.

The following definitions were displayed to respondents in a pop-up box if clicked for more information.

- What is a home saliva sample? A home saliva sample would involve you spitting in a tube and sending it to a certified laboratory.
- What is a home throat swab? A home throat swab would involve you using a throat swab and sending it into a certified laboratory.
- What is a drive-through site? A drive-through site for throat swab would involve your traveling to a drive-through facility in your car to have a healthcare worker collect the swab.
- What is a laboratory throat swab? A laboratory throat swab would involve your traveling to a laboratory facility in a clinic or private laboratory to have a healthcare worker collect the swab.

As part of follow up care:*

	Strongly Agree	Agree	Undecided	Disagree	Strongly Disagree
I would be willing to collect a saliva sample for a home test as part of follow-up care.	◯	◯	◯	◯	◯
I would be willing to use a home throat swab test as part of follow-up care.	◯	◯	◯	◯	◯
I would be willing to go to a drive-through site for throat swab test as part of follow-up care.	◯	◯	◯	◯	◯
I would be willing to go to a clinic for a lab-collected throat swab test as part of follow-up care.	◯	◯	◯	◯	◯

COVID-19 Home Test Perceptions Part 4

Compared to testing in a drive-through facility, would the availability of a home test kit to take a sample from your own mouth (spit or throat swab) for COVID-19 make you:*

The following definitions were displayed to respondents in a pop-up box if clicked for more information.

- Drive-through facility*:* A drive-through site would involve your traveling to a drive-through facility in your car to have a healthcare worker collect a sample from your mouth.
- Sample from your own mouth: A sample from your mouth would involve you spitting in a tube or using a throat swab.

◯ More likely to participate in a **research study**

◯ About the same likelihood to participate in a **research study**

◯ Less likely to participate in a **research study**

<u>Compared to testing in a laboratory or clinic</u>, would the availability of a home test kit to take a sample from your own mouth (spit or throat swab) for COVID-19 make you:*

The following definitions were displayed to respondents in a pop-up box if clicked for more information.

- <u>Laboratory or clinic</u>*:* A laboratory test would involve your traveling to a laboratory facility in a clinic or private laboratory to have a healthcare worker collect a sample from your mouth.
- <u>Sample from your own mouth</u>: A sample from your mouth would involve you spitting in a tube or using a throat swab.

○ More likely to participate in a **research study**

○ About the same likelihood to participate in a **research study**

○ Less likely to participate in a **research study**

COVID-19 Home Test Perceptions Part 5

<u>Compared to testing in a drive-through facility</u>, would the availability of a home test kit to take a sample from your own mouth (spit or throat swab) for COVID-19 make you:*

The following definitions were displayed to respondents in a pop-up box if clicked for more information.

- <u>Drive-through facility</u>*:* A drive-through site would involve your traveling to a drive-through facility in your car to have a healthcare worker collect a sample from your mouth.
- <u>Sample from your own mouth</u>: A sample from your mouth would involve you spitting in a tube or using a throat swab.

○ More likely to seek COVID19 testing if you are **feeling ill**

○ About the same likelihood to seek COVID19 testing if you are **feeling ill**

○ Less likely to seek COVID19 testing if you are **feeling ill**

<u>Compared to testing in a laboratory or clinic</u>, would the availability of a home test kit to take a sample from your own mouth (spit or throat swab) for COVID-19 make you:*

The following definitions were displayed to respondents in a pop-up box if clicked for more information.

- <u>Laboratory or clinic</u>*:* A laboratory test would involve your traveling to a laboratory facility in a clinic or private laboratory to have a healthcare worker collect a sample from your mouth.
- <u>Sample from your own mouth</u>: A sample from your mouth would involve you spitting in a tube or using a throat swab.

○ More likely to seek COVID19 testing if you are **feeling ill**

○ About the same likelihood to seek COVID19 testing if you are **feeling ill**

○ Less likely to seek COVID19 testing if you are **feeling ill**

COVID-19 Home Test Perceptions Part 6

The next questions are about testing for COVID-19 antibodies by using a blood test. Your results would be returned to you electronically and privately.*

The following definitions were displayed to respondents in a pop-up box if clicked for more information.

- What is a home blood test?*:* A home blood test would involve using an automated finger prick device, collecting a blood sample on a specimen card, and mailing in a prepaid mailer to a certified laboratory.
- What is a laboratory blood test?: A laboratory blood test would involve your traveling to a laboratory facility in a clinic or a private lab to have a blood draw similar to a usual doctor's visit.

	Strongly Agree	Agree	Undecided	Disagree	Strongly Disagree
I would be willing to use a **home blood test** as part of a research study.	○	○	○	○	○
I would be willing to go to a clinic for a **lab-collected blood test** as part of a research study.	○	○	○	○	○

Compared to testing in a laboratory or clinic, would availability of a home blood test kit for COVID-19 make you:*

○ More likely to participate in a **research study**

○ About the same likelihood to participate in a **research study**

○ Less likely to participate in a **research study**

Children Intro

Do you have children under age 18 in your household?*

○ Yes

○ No

Page logic: This page will show when question "Do you have children under age 18 in your household?" is "Yes".

Testing Children Part 1

The next questions are about testing YOUR CHILD for COVID-19 by using a saliva sample or a throat swab. Your results would be returned to you electronically and privately.

The following definitions were displayed to respondents in a pop-up box if clicked for more information.

- What is a home saliva sample?: A home saliva sample would involve your child spitting in a tube and sending it to a certified laboratory.
- What is a home throat swab?: A home throat swab would involve you using a throat swab and sending it into a certified laboratory.
- What is a drive-through site?: A drive-through site for throat swab would involve your traveling to a drive-through facility in your car to have a healthcare worker collect the swab.
- What is a laboratory throat swab?: A laboratory throat swab would involve your traveling to a laboratory facility in a clinic or private laboratory to have a healthcare worker collect the swab.

For a research study:*

	Strongly Agree	Agree	Undecided	Disagree	Strongly Disagree
I would be willing to collect a saliva sample for a home test from my child as part of a research study.	○	○	○	○	○
I would be willing to apply a home throat swab test to my child as part of a research study.	○	○	○	○	○
I would be willing to take my child to a drive-through site for throat swab test as part of a research study.	○	○	○	○	○
I would be willing to take my child to a clinic for a lab-collected throat swab test as part of a research study.	○	○	○	○	○

Page logic: This page will show when question "Do you have children under age 18 in your household?" is "Yes".

Testing Children Part 2

The next questions are about testing YOUR CHILD for COVID-19 by using a saliva sample or a throat swab. Your results would be returned to you electronically and privately.

The following definitions were displayed to respondents in a pop-up box if clicked for more information.

- What is a home saliva sample?: A home saliva sample would involve your child spitting in a tube and sending it to a certified laboratory.
- What is a home throat swab?: A home throat swab would involve you using a throat swab and sending it into a certified laboratory.
- What is a drive-through site?: A drive-through site for throat swab would involve your traveling to a drive-through facility in your car to have a healthcare worker collect the swab.
- What is a laboratory throat swab?: A laboratory throat swab would involve your traveling to a laboratory facility in a clinic or private laboratory to have a healthcare worker collect the swab.

To learn if my child has COVID-19:*

	Strongly Agree	Agree	Undecided	Disagree	Strongly Disagree
I would be willing to collect a saliva sample for a home test from my child to learn if they have COVID-19.	○	○	○	○	○
I would be willing to apply a home throat swab test to my child to learn if they have COVID-19.	○	○	○	○	○
I would be willing to take my child to a drive-through site for throat swab test to learn if they have COVID-19.	○	○	○	○	○
I would be willing to take my child to a clinic for a lab-collected throat swab test to learn if they have COVID-19.	○	○	○	○	○

Page logic: This page will show when question "Do you have children under age 18 in your household?" is "Yes".

Testing Children Part 3

The next questions are about testing YOUR CHILD for COVID-19 by using a saliva sample or a throat swab. Your results would be returned to you electronically and privately.

The following definitions were displayed to respondents in a pop-up box if clicked for more information.

- What is a home saliva sample?: A home saliva sample would involve your child spitting in a tube and sending it to a certified laboratory.
- What is a home throat swab?: A home throat swab would involve you using a throat swab and sending it into a certified laboratory.
- What is a drive-through site?: A drive-through site for throat swab would involve your traveling to a drive-through facility in your car to have a healthcare worker collect the swab.
- What is a laboratory throat swab?: A laboratory throat swab would involve your traveling to a laboratory facility in a clinic or private laboratory to have a healthcare worker collect the swab.

For follow-up care:*

	Strongly Agree	Agree	Undecided	Disagree	Strongly Disagree
I would be willing to collect a saliva sample for a home test from my child as part of follow-up care.	○	○	○	○	○
I would be willing to apply a home throat swab test to my child as part of follow-up care.	○	○	○	○	○
I would be willing to take my child to a drive-through site for throat swab test as part of follow-up care.	○	○	○	○	○
I would be willing to take my child to a clinic for a lab-collected throat swab test as part of follow-up care.	○	○	○	○	○

Page logic: This page will show when question "Do you have children under age 18 in your household?" is "Yes".

Testing Children Part 4

<u>Compared to testing your child in a drive-through facility</u>, would the availability of a home test kit to take a sample from your child's mouth (spit or throat swab) for COVID-19 make you:*

The following definitions were displayed to respondents in a pop-up box if clicked for more information.

- <u>Drive-through facility</u>: A drive-through site would involve your traveling to a drive-through facility in your car to have a healthcare worker collect a sample from your child's mouth.
- <u>Sample from your child's mouth</u>: A sample from your child's mouth would involve your child spitting in a tube or using a throat swab.

- More likely to have **<u>your child</u>** participate in a **research study**
- About the same likelihood to have **<u>your child</u>** participate in a **research study**
- Less likely to have **<u>your child</u>** participate in a **research study**

<u>Compared to testing your child in a laboratory or clinic</u>, would the availability of a home test kit to take a sample from your child's mouth (spit or throat swab) for COVID-19 make you:*

The following definitions were displayed to respondents in a pop-up box if clicked for more information.

- <u>Laboratory or clinic</u>: A laboratory test would involve your traveling to a laboratory facility in a clinic or private laboratory to have a healthcare worker collect a sample from your child's mouth.
- <u>Sample from your child's mouth</u>: A sample from your child's mouth would involve your child spitting in a tube or using a throat swab.

- More likely to have **<u>your child</u>** participate in a **research study**
- About the same likelihood to have **<u>your child</u>** participate in a **research study**
- Less likely to have **<u>your child</u>** participate in a **research study**

Page logic: This page will show when question "Do you have children under age 18 in your household?" is "Yes".

Testing Children Part 5

<u>Compared to testing your child in a drive-through facility</u>, would the availability of a home test kit to take a sample from your child's mouth (spit or throat swab) for COVID-19 make you:*

The following definitions were displayed to respondents in a pop-up box if clicked for more information.

- <u>Drive-through facility</u>: A drive-through site would involve your traveling to a drive-through facility in your car to have a healthcare worker collect a sample from your child's mouth.
- <u>Sample from your child's mouth</u>: A sample from your child's mouth would involve your child spitting in a tube or using a throat swab.

- More likely to have **<u>your child</u>** get COVID19 testing if they are **feeling ill**

○ About the same likelihood to have **<u>your child</u>** get COVID19 testing if they are **feeling ill**

○ Less likely to have **<u>your child</u>** get COVID19 testing if they are **feeling ill**

<u>Compared to testing your child in a laboratory or clinic</u>, would the availability of a home test kit to take a sample from your child's mouth (spit or throat swab) for COVID-19 make you:*

The following definitions were displayed to respondents in a pop-up box if clicked for more information.

- <u>Laboratory or clinic</u>: A laboratory test would involve your traveling to a laboratory facility in a clinic or private laboratory to have a healthcare worker collect a sample from your child's mouth.
- <u>Sample from your child's mouth</u>: A sample from your child's mouth would involve your child spitting in a tube or using a throat swab.

○ More likely to have **<u>your child</u>** get COVID19 testing if they are **feeling ill**

○ About the same likelihood to have **<u>your child</u>** get COVID19 testing if they are **feeling ill**

○ Less likely to have **<u>your child</u>** get COVID19 testing if they are **feeling ill**

Page logic: This page will show when question "Do you have children under age 18 in your household?" is "Yes".

Testing Children Part 6

The next questions are about testing <u>YOUR CHILD</u> for COVID-19 antibodies by using a <u>blood test</u>. Your results would be returned to you electronically and privately. *

The following definitions were displayed to respondents in a pop-up box if clicked for more information.

- <u>What is a home blood test?</u>: A home blood test would involve using an automated finger prick device, collecting a blood sample on a specimen card, and mailing in a prepaid mailer to a certified laboratory.
- <u>What is a laboratory blood test?</u>: A laboratory blood test would involve your traveling to a laboratory facility in a clinic or a private lab to have a blood draw similar to a usual doctor's visit.

	**Strongly Agree**	**Agree**	**Undecided**	**Disagree**	**Strongly Disagree**
I would be willing to apply a home blood test to my child as part of a research study.	○	○	○	○	○
I would be willing to take my child to a clinic for a lab-collected blood test as part of a research study.	○	○	○	○	○

Demographics: Part 2

Thank you for all of the information you have provided so far. We have just a few last questions.

Our research group is also enrolling for another study about COVID-19. That study is offering a $50 token of appreciation for eligible participants to complete a home specimen collection kit for COVID-19 and a behavioral survey. The study will test the willingness and acceptability to complete and send at-home specimens, and gather input to improve home specimen kit collection instructions.

Are you interested in learning more about this study?*

- Yes
- No

Question logic: This question will show when question "Are you interested in learning more about this study?" is "Yes".

Thank you for your interest. Please provide an email address so we can reach out to you with more information about the specimen collection study.

[ ]

Note: Gender identity question was moved from Part 1 Demographics to Part 2 Demographics on the final day of data collection as part of amendment to increase minority representation in the sample.

What is your current gender identity?*

- Male
- Female
- Transgender female/Trans woman
- Transgender male/Trans man
- Genderqueer
- Other (please specify): [ ]

What is the highest level in school that you completed?

- College, post graduate, or professional school
- Some college, Associate's Degree and/or Technical School
- High school or GED

- Did not finish high school

What was your household income last year from all sources before taxes? (monthly/yearly)

- $0 to $417 (monthly) / $0 to $4,999 (yearly)
- $418 to $833 (monthly) / $5,000 to $9,999 (yearly)
- $834 to $1,250 (monthly) / $10,000 to $14,999 (yearly)
- $1,251 to $1,999 (monthly) / $15,000 to $23,999 (yearly)
- $2,000 to $2,500 (monthly) / $24,000 to $29,999 (yearly)
- $2,501 to $3,333 (monthly) / $30,000 to $39,999 (yearly)
- $3,334 to $4,167 (monthly) / $40,000 to $49,999 (yearly)
- $4,168 to $4,999 (monthly) / $50,000 to $59,999 (yearly)
- $5,000 to $6,250 (monthly) / $60,000 to $74,999 (yearly)
- $6,251 or more (monthly) / $75,000 or more (yearly)
- Don't know

Thank You!

Thank you for taking our survey. Your response is very important to us.

For more information about Coronavirus and how to protect yourself, please visit https://www.cdc.gov/coronavirus/2019-ncov/index.html.
